# A mutation creating an upstream translation initiation codon in *SLC22A5* 5′UTR is a frequent cause of primary carnitine deficiency

**DOI:** 10.1002/humu.23839

**Published:** 2019-07-03

**Authors:** Sacha Ferdinandusse, Heleen te Brinke, Jos P.N. Ruiter, Janet Haasjes, Wendy Oostheim, Henk van Lenthe, Lodewijk IJlst, Merel S. Ebberink, Ronald J.A. Wanders, Frédéric M. Vaz, Hans R. Waterham

**Affiliations:** ^1^ Laboratory Genetic Metabolic Diseases, Amsterdam Gastroenterology and Metabolism Research Institute University of Amsterdam Amsterdam The Netherlands; ^2^ United for Metabolic Diseases The Netherlands

**Keywords:** 5′‐untranslated region, carnitine transport, OCTN2 deficiency, primary or systemic carnitine deficiency

## Abstract

Primary carnitine deficiency is caused by a defect in the active cellular uptake of carnitine by Na^+^‐dependent organic cation transporter novel 2 (OCTN2). Genetic diagnostic yield for this metabolic disorder has been relatively low, suggesting that disease‐causing variants are missed. We Sanger sequenced the 5′ untranslated region (UTR) of *SLC22A5* in individuals with possible primary carnitine deficiency in whom no or only one mutant allele had been found. We identified a novel 5′‐UTR c.‐149G>A variant which we characterized by expression studies with reporter constructs in HeLa cells and by carnitine‐transport measurements in fibroblasts using a newly developed sensitive assay based on tandem mass spectrometry. This variant, which we identified in 57 of 236 individuals of our cohort, introduces a functional upstream out‐of‐frame translation initiation codon. We show that the codon suppresses translation from the wild‐type ATG of *SLC22A5*, resulting in reduced OCTN2 protein levels and concomitantly lower transport activity. With an allele frequency of 24.2% the c.‐149G>A variant is the most frequent cause of primary carnitine deficiency in our cohort and may explain other reported cases with an incomplete genetic diagnosis. Individuals carrying this variant should be clinically re‐evaluated and monitored to determine if this variant has clinical consequences.

## 1 INTRODUCTION

Primary (or systemic) carnitine deficiency (MIM# 212140) is caused by a defect in the active cellular uptake of carnitine by the Na^+^‐dependent organic cation transporter novel 2 (OCTN2; Nezu et al., 1999; Tang et al., 1999). This defect results in urinary carnitine wasting, low serum carnitine levels and decreased intracellular carnitine levels. Because carnitine is essential for the transport of long‐chain fatty acids into the mitochondrion for β‐oxidation, the lack of carnitine results in impaired fatty acid oxidation which affects in particular organs that rely on energy production by fatty acid oxidation such as the heart, skeletal muscle and liver (Houten, Violante, Ventura, & Wanders, 2016; Longo, Frigeni, & Pasquali, 2016). The clinical presentation of primary carnitine deficiency shows great variability ranging from hypoketotic hypoglycemia and hepatic encephalopathy early in life, skeletal‐ and cardiomyopathy later in life, sudden death from cardiac arrhythmia to only fatigue, or no clinical symptoms at all (see for review (Longo, 2016; Magoulas & El‐Hattab, 2012)). The first individuals identified with primary carnitine deficiency all presented with severe clinical symptoms, including acute metabolic decompensation or cardiomyopathy, but because the disorder has been included in many neonatal screening programs worldwide, an increasing number of individuals with milder or no clinical symptoms have been identified. These include asymptomatic mothers with this condition, identified via neonatal screening of their newborn children, who are carriers and picked up due to maternal carnitine deficiency (Longo, 2016; Schimmenti et al., 2007). Although these mothers are usually asymptomatic, they may be at risk for sudden death from arrhythmia (Longo, 2016; Schimmenti et al., 2007).

Primary carnitine deficiency is an autosomal recessive disorder caused by mutations in the OCTN2‐encoding *SLC22A5* gene located on chromosome 5q31 (Nezu et al., 1999; Tang et al., 1999). Over 150 different mutations in *SLC22A5* have been reported (Frigeni et al., 2017; Li et al., 2010). It has been shown that the frequency of nonsense mutations is significantly increased in symptomatic patients and that the residual carnitine‐transport activity is lower in fibroblasts from symptomatic patients than in fibroblasts from asymptomatic women (Rose et al., 2012).

When primary carnitine deficiency is suspected or a newborn is identified via screening, first plasma acylcarnitine analysis, including free carnitine levels, is performed followed by measurements of carnitine‐transport activity in fibroblasts and/or genetic analysis of the *SLC22A5* gene (Longo, 2016; Magoulas & El‐Hattab, 2012). The carnitine‐transport activity assay is reliable for confirming the diagnosis but is only performed in a small number of laboratories throughout the world and requires a skin biopsy being taken. For this reason, in most cases, genetic analysis is performed.

Recently, it was shown in a large study including fibroblasts from 358 subjects with possible primary carnitine deficiency, that carnitine‐transport activity was reduced to 20% or less of normal in fibroblasts of 140/358 subjects. Subsequent Sanger sequence analysis of 95 of the 140 biochemically proven OCTN2‐deficient cells revealed mutations in the coding regions of only 84% of the *SLC22A5* alleles (Frigeni et al., 2017).

We also observed a relatively low diagnostic yield with genetic testing of the *SLC22A5* gene. Sanger sequencing of the coding regions of the *SLC22A5* gene in 236 individuals with possible primary carnitine deficiency revealed bi‐allelic mutations in 133 individuals (56.4%). In the remaining individuals, we only identified one heterozygous (69 individuals; 29.2%) or no mutation (34 individuals; 14.4%).

Here we report the identification and characterization of a 5′ untranslated region (UTR) variant in *SLC22A5*, c.‐149G>A, which introduces a functional upstream out‐of‐frame translation initiation codon. We show that this codon suppresses translation from the wild‐type AUG of *SLC22A5*, resulting in reduced OCTN2 protein levels and concomitantly lower OCTN2 transport activity explaining the carnitine deficiency in patients harboring this variant. We identified this variant in 57 individuals in whom we previously identified only one or no mutation in the coding region of *SLC22A5*.

## 2 METHODS

### 2.1 PCR and sequence analysis

For standard Sanger sequencing of *SLC22A5*, genomic DNA was isolated from primary skin fibroblasts using the NucleoSpin Tissue genomic kit (Macherey‐Nagel). The coding regions including the exon/intron boundaries of the *SLC22A5* gene were amplified by PCR using 10 primer pairs tagged with a ‐21M13 (5′‐TGTAAAACGACGGCCAGT‐3′) sequence or M13rev (5′‐CAGGAAACAGCTATGACC‐3′) sequence, respectively. PCR fragments were sequenced using “‐21M13,” “M13rev” primers by means of BigDye Terminator v1.1 Cycle Sequencing Kits (Applied Biosystems) and analyzed on 3130 x 1 or 3730 x 1 DNA analyzers (Applied Biosystems), following the manufacturer′s protocol. Sequence reads (electropherograms) were analyzed using CodonCode Aligner software package (CodonCode Corporation) and compared to *SLC22A5* reference sequence GenBank: NM_003060.3 (GRCh38, chr5).

### 2.2 Expression constructs

To allow comparison of translation initiation efficiency between the wild‐type c.‐149G allele and the mutated c.‐149A allele, the entire 5′UTR plus the first 15 (i.e., c.‐264C_c.15C) or 16 (i.e., c.‐264C_c.16G) coding nucleotides of exon 1 of the WT *SLC22A5* gene were cloned 5′ of the NanoLuc luciferase open reading frame (ORF), lacking its own ATG start codon and cloned in pcDNA5/FRT (GenScript Biotech). The c.‐149G>A mutation was introduced using the QuikChange II Site‐Directed Mutagenesis Kit (Agilent Technologies, Santa Clara), following the manufacturer′s protocol. The c.‐264C_c.15C constructs have the original ATG in frame with the NanoLuc luciferase ORF, whereas the c.‐264C_c.16G constructs have the ATG introduced by c.‐149G>A in frame with the NanoLuc luciferase ORF. Figure 2 shows a graphical presentation of the four different constructs.

### 2.3 Transfection and NanoLuc luciferase assay

HeLa FlpIn cells were cotransfected with pOG44 and the different NanoLuc luciferase pcDNA 5/FRT reporter constructs using JetPrime (Strasbourg, France). After transfection, cells were cultured in DMEM (Gibco) supplemented with 10% FBS (Bodinco), 100 U/ml penicillin, 100 mg/ml streptomycin (LifeTechnologies), 250 ng/ml Fungizone (LifeTechnologies) and, for selection of transfected cells, Hygromycin (Sigma Aldrich) in a humidiﬁed atmosphere of 5% CO_2_ at 37°C. Single cell colonies were obtained after serial dilution in 96‐well plates. Expanded cell colonies were plated in 96 wells plate for 24 hr after which NanoLuc luciferase activity (Nano‐Glo Luciferase assay, Promega) was measured according to the manufacturer′s protocol using the spectrophotometer Infinite M200 Pro (Tecan).

### 2.4 Carnitine transport assay

Control and patient primary fibroblasts (equivalent to approximately 20 µg protein/well) were cultured in quadruplicate in 24‐well tissue culture plates (Costar) in HAM F‐10 (Gibco) supplemented with 10% Fetal Bovine serum (Bodinco), 100 U/ml penicillin, 100 mg/ml streptomycin (LifeTechnologies), and 250 ng/ml Fungizone (LifeTechnologies) in a humidiﬁed atmosphere of 5% CO_2_ at 37°C for 1 week. The cells were washed three times with phosphate‐buffered saline (PBS; room temperature) and incubated in Dulbecco′s PBS supplemented with 5 mM glucose, 1 mg/ml bovine serum albumin and 5 µM *D*_3_‐carnitine with and without 0.625 mM of mersalyl acid (Sigma Aldrich) at 37°C 5% CO_2_. After 2 hr, the cells were washed with ice‐cold PBS and the reaction was stopped by adding ethanol supplemented with 25 pmol *D*_*9*_‐carnitine per well as an internal standard. Cells were incubated for 1 hr at −20°C. The ethanol was taken from the cells and evaporated under a stream of nitrogen. The residue was dissolved in 50 µl of 0.1% heptafluorobutyric acid (HFBA; Thermo Scientific, Waltham). Unlabeled, D_3_ and D_9_‐carnitine were measured using UPLC‐MS. The UPLC‐MS system consisted of an Acquity SDS liquid chromatography system coupled to a Premier‐XE mass spectrometer (Waters). Ten microliters of sample was loaded on Waters Acquity UPLC BEH C18, 1.7 µm, 2.1 × 100 mm, maintained at 50°C, equilibrated with 0.1% HBFA and eluted using methanol. D_3_‐carnitine was quantified in the positive ion mode using D_9_‐carnitine as internal standard using a calibration curve of unlabeled carnitine. The amount of D_3_‐carnitine was used to calculate the carnitine‐transport activity.

## 3 RESULTS

### 3.1 Analysis for *SLC22A5* noncoding mutations

Because the finding of single heterozygous *SLC22A5* mutations in a series of patients biochemically confirmed with primary carnitine deficiency suggested the presence of a second, noncoding mutation in trans rather than involvement of a different gene, we searched for mutations outside the coding region of *SLC22A5*. First, we Sanger sequenced *SLC22A5* cDNA prepared from messenger RNA isolated from primary fibroblasts of 12 non‐related patients with biochemically confirmed primary carnitine deficiency in whom no (2 patients) or only one heterozygous mutation (10 patients) had been identified. Except for heterozygosity of the known mutation in the 10 patients, we did not identify novel variants or indications for large deletions, nonsense‐mediated decay or exon skipping. We also did not observe allelic expression imbalance promoting expression of the allele containing the heterozygous mutation, a phenomenon we recently reported as underlying cause for Zellweger spectrum disorder (Falkenberg et al, 2017). When we next sequenced the 264‐bp‐long 5′UTR sequence of the *SLC22A5* gene, we noted that all patients heterozygous for a known mutation were also heterozygous for a GRCh38:5:132369824:G:A; NM_003060.3:c.‐149G>A variant, while two patients, in whom no mutations had been found, were homozygous for this variant. This c.‐149G>A variant is not found in the ExAC browser database but is mentioned in the gnomAD browser database of the Exome Aggregation Consortium with an overall allele frequency of 0.001278 in 31306 genomes (Lek et al., 2016).

**Figure 1:**
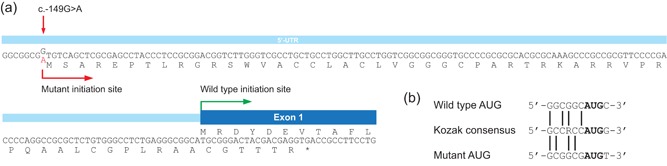
Molecular characterization of *SLC22A5* c.‐149G>A variant. (a) Schematic representation of 5′UTR and exon 1 of *SLC22A5*. The c.‐149G>A variant introduces a novel translation initiation site. The predicted mutant protein is shown and the partial wild‐type protein. The mutant protein is predicted to result in a premature termination codon in exon 1. (b) Comparison of the sequence context surrounding the wild‐type and mutant AUG (created by c.‐149G>A variant) for the presence of a KOZAK sequence

Interestingly, the c.‐149G>A variant introduces a novel putative AUG translation initiation codon 149 nucleotides upstream of the normal AUG start codon (at position c.1). Translation from this upstream AUG would create a novel upstream ORF of 56 codons, which is out of frame with the normal ORF and terminates at nucleotide c.20 (see Figure 1). The flanking sequences of the c.‐149G>A–introduced upstream AUG and the wild‐type AUG each show a 4/7 nucleotide match with the Kozak consensus sequence (Kozak, 1986; see Figure 1), but the calculated reliability score of the Kozak sequence surrounding the mutant AUG was lower than of the wild‐type AUG (0.13 vs. 0.90; Salamov, Nishikawa, & Swindells, 1998).

### 3.2 Functional assessment of the c.‐149G>A variant

**Figure 2:**
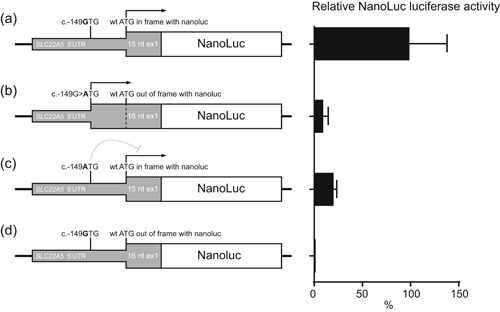
Functional characterization of SLC22A5 c.‐149G>A variant. HeLa cells were transfected with constructs of the 5′‐UTR and the first 15 (i.e. c.1‐264C_c.15C) (a,c) or 16 (i.e. c.1‐264C_c.16) (b,d) coding nucleotides of exon 1 of SLC22A5 fused to NanoLuc. Left: graphical presentations of the constructs used (a–d). The positions and possible use (arrow) of the wild‐type initiation codon and the novel initiation codon introduced by the c.‐149G>A variant are indicated. Construct a and c have the original ATG in frame with the NanoLuc luciferase open reading frame, whereas construct b has the ATG introduced by c.1‐49G>A in frame with NanoLuc luciferase. Right: relative NanoLuc luciferase activity presented as % of the mean activity of two independently transfected and analyzed wild‐type constructs (a)

To determine if the 5′‐UTR c.‐149G>A variant introduces a functional translation initiation codon that is recognized and used in vivo, we transfected HeLa cells with different reporter constructs generated from pcDNA3 containing the NanoLuc luciferase ORF preceded by wild‐type or mutant 5′UTR sequences and the first 15 or 16 nucleotides of exon 1 (see Figure 2). Cells transfected with the reporter constructs in which either the 5′UTR containing the wild‐type AUG or the 5′UTR containing the mutant AUG is in frame with NanoLuc luciferase, both showed luciferase activity, indicating that the mutant AUG can be used for translation initiation (see Figure 2). However, the luciferase activity in the cells transfected with the 5′UTR containing the mutant AUG in frame with NanoLuc luciferase was much lower than the activity in the cells transfected with the wild‐type 5′UTR containing the wild‐type AUG in frame with NanoLuc luciferase (11% compared with the wild‐type AUG). Moreover, cells transfected with the 5′UTR containing both the mutant and the wild‐type AUG and with the wild‐type AUG in frame with NanoLuc luciferase also showed reduced luciferase activity. Taken together, these findings indicate that the novel AUG created by the 5′‐UTR c.‐149G>A variant creates a functional, but weaker, translation initiation codon, which suppresses translation from the wild‐type AUG of *SLC22A5*.

### 3.3 Functional consequence of the c.‐149G>A variant

**Figure 3:**
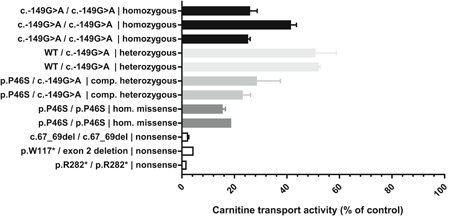
Functional consequence of *SLC22A5* c.‐149G>A variant. The carnitine‐transport activity was measured in fibroblasts of control subjects and fibroblasts with different mutations in *SLC22A5*. The genotype of the different cell lines is indicated and the activity is expressed as a percentage of the mean activity of the two control cell lines measured in the same experiment. All measurements were done in duplicate and all cell lines were analyzed in two independent experiments minimally, except for one cell line (*) which was only analyzed in one experiment

The functional expression studies indicate that the presence of the upstream AUG causes a reduction in the synthesis of OCTN2. Unfortunately, this cannot be assessed by immunoblot analysis in homogenates of patient fibroblasts due to the lack of specific antibodies that recognize OCTN2 in fibroblasts. As an alternative, we studied the consequence of the c.‐149G>A variant for the carnitine‐transport activity by OCTN2 in fibroblasts of patients homozygous for this variant, or compound heterozygous for this variant and a known pathogenic mutation in *SLC22A5*. For this purpose, we developed a novel sensitive method using stable isotope‐labeled carnitine (D_3_‐carnitine) as a substrate to determine the amount of D_3_‐carnitine taken up by skin fibroblasts of the patients (i.e., carnitine transport) by means of tandem mass spectrometry. For comparison, we used control fibroblasts, patient fibroblasts with severe truncating mutations in *SLC22A5* and fibroblasts with the known mild mutation c.136C>T (p.P46S). All cell lines with the c.‐149G>A variant (either homozygous or compound heterozygous in combination with the c.136C>T mutation) had reduced but considerable residual carnitine‐transport activity compared to the activity in control fibroblasts (see Figure 3). On average, we found 31% of the activity in control lines in homozygotes and 26% in compound heterozygotes. Fibroblasts from patients with severe mutations showed an almost complete deficiency of OCTN2 activity (2% of the activity in controls). The residual OCTN2 activity in cells homozygous for the c.‐149G>A variant was somewhat higher than in cells homozygous for the known mild c.136C>T mutation, which also displayed residual OCTN2 activity. Cells heterozygous for a single c.‐149G>A variant displayed on average 52% of control activity. These combined results confirm that the c.‐149G>A variant results in reduced carnitine‐transport activity, most likely due to a lower expression of the OCTN2 protein.

### 3.4 Frequency of c.‐149G>A variant

Having established that the c.‐149G>A variant causes a functional defect of OCTN2 activity, we reanalyzed the *SLC22A5* gene of the individuals, in whom we previously found only one heterozygous (69 individuals) or no mutations (34 individuals) in *SLC22A5,* for the presence of the variant. We identified the variant in 57 individuals, which amounts to 24.2% of our cohort comprising 236 individuals with possible primary carnitine deficiency. Of these 57 individuals, 16 were heterozygous, 33 compound heterozygous, and 8 homozygous. Together this results in an allele frequency of 13.8% for the c.‐149G>A variant in our cohort. For comparison, we identified the most common mutation reported so far for primary carnitine deficiency, c.136C>T (p.(Pro46Ser); Magoulas & El‐Hattab, 2012), in 50 individuals (21.1%) with an allele frequency of 11.2%.

Of the 236 individuals in our cohort, 88 were analyzed as follow‐up to a positive newborn screening result. Of these, 33 individuals (37.5%) carried one or two copies of the c.‐149G>A variant while 28 individuals (31.8%) carried one or two copies of the c.136C>T (p.(Pro46Ser)) variant.

## 4 DISCUSSION

Primary carnitine deficiency is screened for in many NBS programs worldwide. Early and proper diagnosis of patients with this defect is very important so that patients can be treated with carnitine supplementation. If left untreated, patients can develop severe clinical symptoms like hypoketotic hypoglycemic early in life and/or skeletal or cardiac myopathy, or risk dying from sudden death. In most cases, diagnosis is done by analysis of acylcarnitines in blood/plasma followed by genetic analysis. If results are inconclusive, e.g., in case of finding DNA variants of unknown significance, OCTN2 activity can be measured in fibroblasts which can confirm or exclude the diagnosis. A wide variety of mutations have been identified in the OCTN2‐encoding gene *SLC22A5*. However, the mutation detection frequency has always been lower than for most autosomal recessive inherited metabolic diseases in published reports as well in our diagnostic experience. In a recent study, it was reported that 16% of the mutant alleles in cells of 95 subjects with a reduced carnitine‐transport activity, could not be identified by Sanger sequencing and deletion/duplication analysis of all 10 exons of the *SLC22A5* gene and flanking regions (Frigeni et al., 2017).

We now identified a c.‐149G>A variant in the 5′UTR of *SLC22A5*, which introduces a novel AUG translation initiation codon upstream of the normal AUG start codon that causes suppression of translation from the normal AUG, resulting in reduced carnitine‐transport activity in fibroblasts harboring this variant. This variant is not found in the ExAC browser database but is mentioned in the gnomAD browser database of the Exome Aggregation Consortium albeit with a low overall allele frequency of 0.001278 in 31306 genomes (http://gnomad.broadinstitute.org/variant/5-131705516-G-A, accessed November 2018). This suggests that this mutation may be more frequent, but not covered by standard WES, either because it is located in the noncoding 5′UTR region not well covered by WES or due to the GC‐rich regions in exon 1 that impede its analysis, or both. Indeed, we identified the variant in 65 of the 472 analyzed alleles of 236 individuals (from different nationalities) with possible primary carnitine deficiency, showing that with an allele frequency of 24.2% this variant is the most frequent cause of primary carnitine deficiency in our cohort. The majority of the individuals carrying the c.‐149G>A variant in our cohort includes children or mothers of children picked up via NBS, based on low blood carnitine levels. For comparison, the c.136C>T (p.P46S) mutation, which is known to be very frequent in asymptomatic individuals picked up via NBS (Li et al., 2010; Magoulas & El‐Hattab, 2012), has an allele frequency of 21.1% in our cohort.

To study the consequence of DNA variants on OCTN2 activity in patients fibroblasts we developed a novel sensitive assay based on tandem mass spectrometry. This assay is more sensitive than the currently used radiochemical assays and allows accurate measurement of low residual activities. Using this assay, we found that the residual OCTN2 activity in fibroblasts homozygous for the c.‐149G>A variant was higher (31%) than the activity in fibroblasts homozygous for the c.136C>T (p.P46S) mutation (17%).

As far as we are aware, individuals carrying the c.‐149G>A variant never experienced any severe clinical symptoms that could be related to primary carnitine deficiency, such as severe brain dysfunction, cardiomyopathy, muscle weakness, or hypoglycemia. However, it will require clinical re‐evaluation and long‐term monitoring to determine if this variant has any clinical consequences.

Mutations in the 5′UTR as cause of disease has been described for different diseases, including autosomal recessive metabolic disorders (Barbosa, Peixeiro, & Romão, 2013; Fu et al., 2015; Semler et al., 2012; von Bohlen et al., 2017; Willemsen et al., 2017) and thus should be considered in patients with autosomal recessive disease in whom only one heterozygous mutation has been found. Indeed, such disease‐causing mutations may be more frequent than previously assumed due to the fact that most clinical DNA testing specifically focuses on the coding regions of genes.

## CONFLICT OF INTERESTS

The authors declare that they have no conflict of interests.

## DATA AVAILABILITY

The novel mutation has been submitted to the Leiden Open Variant Database (LOVD): https://databases.lovd.nl/shared/genes/SLC22A5.
